# Improvements in gait characteristics after intensive resistance and functional training in people with dementia: a randomised controlled trial

**DOI:** 10.1186/1471-2318-14-73

**Published:** 2014-06-12

**Authors:** Michael Schwenk, Tania Zieschang, Stefan Englert, Gurtej Grewal, Bijan Najafi, Klaus Hauer

**Affiliations:** 1 Department of Geriatric Research, Bethanien-Hospital/ Geriatric Center at the University of Heidelberg, Heidelberg, Germany; 2 Department of Surgery, Interdisciplinary Consortium on Advanced Motion Performance (iCAMP), College of Medicine, University of Arizona, 1656 E Mabel Street, Tucson, Arizona 85724, USA; 3 Arizona Center on Aging, University of Arizona, Tucson, USA; 4 Institute of Medical Biometry and Informatics, University of Heidelberg, Heidelberg, Germany

## Abstract

**Background:**

Preventing and rehabilitating gait disorders in people with dementia during early disease stage is of high importance for staying independent and ambulating safely. However, the evidence gathered in randomized controlled trials (RCTs) on the effectiveness of exercise training for improving spatio-temporal gait parameters in people with dementia is scarce. The aim of the present study was to determine whether a specific, standardized training regimen can improve gait characteristics in people with dementia.

**Methods:**

Sixty-one individuals (mean age: 81.9 years) with confirmed mild to moderate stage dementia took part in a 3-month double-blinded outpatient RCT. Subjects in the intervention group (IG) received supervised, progressive resistance and functional group training for 3 months (2 times per week for two hours) specifically developed for people with dementia. Subjects in the control group (CG) conducted a low-intensity motor placebo activity program. Gait characteristics were measured before and after the intervention period using a computerized gait analysis system (GAITRite®).

**Results:**

Adherence to the intervention was excellent, averaging 91.9% in the IG and 94.4% in the CG. The exercise training significantly improved gait speed (P < 0.001), cadence (P = 0.002), stride length (P = 0.008), stride time (P = 0.001), and double support (P = 0.001) in the IG compared to the CG. Effect sizes were large for all gait parameters that improved significantly (Cohen’s d: 0.80-1.27). No improvements were found for step width (P = 0.999), step time variability (P = 0.425) and Walk-Ratio (P = 0.554). Interestingly, low baseline motor status, but not cognitive status, predicted positive training response (relative change in gait speed from baseline).

**Conclusion:**

The intensive, dementia-adjusted training was feasible and improved clinically meaningful gait variables in people with dementia. The exercise program may represent a model for preventing and rehabilitating gait deficits in the target group. Further research is required for improving specific gait characteristics such as gait variability in people with dementia.

**Trial registration:**

ISRCTN49243245

## Background

Gait deficits are among the leading risk factors for falling in both community dwelling and institutionalized older adults [1,2]. Beside common factors for gait deficits such as arthritis, foot problems or stroke, dementia can have a considerable impact on gait performance. A systematic review reported decreased walking speed and step length, increased double limb support duration and gait variability in people with dementia when compared with healthy controls [3]. Furthermore, walking speed decreases as the disease progresses. Gait deficits occur earlier in vascular dementia whereas AD patients gait is affected during moderate-severe stage [3,4].

Preventing and rehabilitating gait disorders in people with dementia during early disease stage is of high importance for staying independent and ambulating safely [5]. Even relatively mild gait impairments may still have a significant impact on functional mobility. For example, reductions in walking speed can impede one’s ability to navigate between two points in a timely manner. This may result in difficulty crossing a street, incontinence accidents, or it may cause people to rush more than usual and may further escalate their instability and risk of falling [5,6].

Controversial results have been reported for the effect of exercise training on gait performance in people with dementia. Some studies did not report significant effects [7-9]. Others found significant improvements in gait speed [10-13], although effect sizes in most of these studies were small [14,15], limiting the clinical meaningfulness of results. Potential causes for the lack of the effectiveness of training interventions include non-specific intervention strategies; insufficient intensity, duration and standardization of training; and lack of specific approaches toward patients with dementia as discussed in systematic reviews [14-17]. The specific factors affecting training response were often not reported in studies [14-17]. Many exercise trials have methodological deficits such as small sample sizes, poorly described randomisation methods, incomparability of study groups at baseline, or insufficient diagnosis of dementia [14-16]. Importantly, most studies have used subjective (e.g. Performance Oriented Mobility Assessment, POMA [11,18,19]) or semi-objective gait measures (stopwatch [7,8,10-13]) only. These measures do not provide spatio-temporal gait parameters beyond speed and are observer-dependent. High quality exercise trials using both objective computerized gait analysis and established exercise programs for providing sound evidence for the positive effect of exercise training on gait characteristics in people with dementia are lacking.

The aim of the present study was to evaluate the effect of a 3-month intensive progressive resistance and functional training program on gait characteristics in people with confirmed mild to moderate dementia. A second aim was to analyse whether cognitive function and/or other factors were associated with training response.

## Methods

### Study design

The study was designed as a double-blinded, randomized, controlled intervention trial. Neither the testers nor the participants were aware of group identity. The study was approved by the ethic committee of the Medical Department of the University of Heidelberg. The trial was registered at http://www.controlled-trials.com (ISRCTN49243245). The results of the parent study focusing on strength and functional performances as quantified by semi-objective (stopwatch) performance-based tests have been published previously [20]. The specific exercise effects on gait characteristics as measured by computerized spatial and temporal gait measurement (GAITRite® system) in a subsample of 61 participants (out of 122 recruited for the parent study) are presented in this paper. As the electronic GAITRite® system was available only during the second half of the parent study, the gait analysis was conducted in a subsample of consecutively recruited participants (participant 62–122) of the parent study.

### Study population

Individuals were consecutively recruited during rehabilitation at a geriatric hospital (Bethanien-Hospital/Geriatric Centre at the University of Heidelberg, Germany) or from outpatient nursing care services from March 2007 to January 2008. Eligible subjects were screened for cognitive function by the Mini-Mental State Examination (MMSE) [21]. In individuals meeting the inclusion criteria for cognitive impairment (MMSE scores 17–26), a dementia diagnosis was confirmed according to international standards [22,23], based on medical history, clinical examination, cerebral imaging, and neuropsychological testing (Consortium to Establish a Registry for Alzheimer’s Disease [24]; Trail Making Test [25]). Further inclusion criteria were age 65 and older; ability to walk 10 metres without walking aid; no uncontrolled or terminal neurologic, cardiovascular, metabolic, or psychiatric disorder; residence within 15 kilometres of the study centre; written informed consent; approval by the treating physician and the legal guardian (if appointed). Subjects meeting the inclusion criteria were randomly assigned to the intervention group (IG) or the control group (CG) after baseline testing at the end of ward rehabilitation. Subjects were assigned to their treatment using the urn design [26] (numbered containers), stratified according to sex and location of recruitment (hospitalized vs. other) The sequence was concealed until interventions were assigned after baseline measurement. A person unrelated to the study performed the randomization procedure and assigned participants to their study group.

### Intervention

The IG underwent a regimen of progressive resistance and functional training in groups of four to six participants for 3 months (2 hours, twice a week) supervised by a qualified instructor as described previously [20,27,28]. Resistance training was targeted at functionally relevant muscle groups at a submaximal intensity (70-80% of one repetition maximum (1-RM)). As the training progressed, the applied weight was continuously increased as necessary to keep the individual within the target range of 70-80% of the 1RM. The 10-RM test was used in order to adjust intensity of resistance training during the intervention period.

The functional training focused on basic activity of daily living (ADL)-related motor functions including sitting down and standing up from a chair, standing (static and dynamic postural control) and walking. When participants were stable in basic walking over a distance of 10 metres they progressed to advanced levels of walking exercise. Complexity and challenge of tasks were progressively increased by using goal-oriented progressively difficult stepping and walking patterns to promote the timing and coordination of walking. Walking patterns progressed by altering speed, amplitude (e.g., narrowing oval width), or accuracy of performance (e.g., without straying from the desired path) and then to complex patterns such as walking past other walkers and combined upper extremity tasks such as carrying, bouncing, or tossing a ball.

Trainers used specific strategies to promote exercise in people with dementia as described by Oddy et al. [29]. This included communication strategies such as speaking slowly and clearly with instructions repeated several times. Simple direct requests (‘Mrs Brown, please walk to the window’) were used rather than indirect requests (‘Mrs Brown, can you walk to the window for me?’). Tactile and rhythmic cues were provided to ensure correct execution of movements. Much attention was focussed on emotional aspects such as reassurance and empathy towards each participant as is described in dementia-care guidelines [30].

All participants assigned to the CG met two times per week for 1 hour of supervised motor placebo group training. Typical activities were flexibility exercise, calisthenics, low-intensity training with hand-held weights, and ball games while seated. Participants were blinded because they had not been informed of the different effectiveness of the two training regimens, which prevented expectations of the training effects.

### Measurements

Measurements were performed before randomization (T1) and at the end of the intervention period (T2). All measurements were performed by validated, established tests. Training adherence was documented as percentage of training sessions successfully performed by each participant.

#### Clinical characteristics

Clinical characteristics including medication (number), comorbidity (Cumulative Illness Rating Scale, CIRS) [31], and social status were documented from patient charts. Screening for depression (Geriatric Depression Scale, GDS, 30-item version) [32] and falls during the previous year were documented by standardized interviews. Functional status was quantified by the Barthel Index [33], the Performance Oriented Mobility Assessment (POMA) [34], and lower extremity strength as measured by the 1-RM as achieved in a leg-press training machine (Kaphingst, Lahntal, Germany).

#### Gait performance

Gait performance was measured by temporal and spatial gait variables (speed, cadence, stride length, stride time, double support [as percentage of stride time], step width, step time variability, and Walk-Ratio defined as step length/cadence-ratio [35]) using a GAITRite® system (CIR Systems Inc., Havertown, PA). The GAITRite® is an electronic gait analysis system (4.9 meter length) based on embedded pressure sensors which demonstrated high validity relative to a 3-dimensional motion analysis system [36]. Subjects were instructed to walk as fast as possible but safely. Participants started walking 2 meters prior to reaching the electronic walkway and stopped 2 meters beyond it for measuring steady-state walking [37]. Each participant performed two walking trials. The mean values of both walks were used for statistical analysis.

Sample size was calculated for increase in gait speed during the training intervention period using results of a previous study [27]. Based on an effect size of d = 1.1, statistical power of 90%, a significance level of .05, and a drop-out rate of 25%, a sample size of 48 participants was needed to verify a significant intervention effect.

### Statistical analysis

Unpaired t-tests and Chi-square-tests were used for baseline comparison according to the scale of the investigated variable. Primary study endpoint was gait speed; all other gait variables were secondary outcome measures. Analysis of covariance (ANCOVA) was used to compare the effect of the intervention on gait variables at follow-up adjusting for baseline values. Effect sizes were calculated as Cohen’s d (adjusted mean treatment difference/pooled standard deviation). A Cohen’s d of 0.2 was considered as small, 0.5 as medium, and 0.8 as large [38].

Univariate linear regression analyses were performed to delineate predictive factors of training response (relative pre- to post changes in gait speed) for the IG. Variables included baseline cognitive parameters (MMSE, Trail-Making Test, CERAD subtests including early and delayed recall, recall of symbols, discrimination, verbal fluency, visuoconstructive abilities), age, gender, ADL-status (Barthel Index), comorbidity (CIRS), depression (GDS), baseline motor variables (POMA, lower extremity strength), and adherence to the intervention. Significant correlates at P-values of 0.1 or less were retained for a multivariate regression model (stepwise backward) to identify independent predictive variables of training response. Influences of variables are given as regression coefficients β and general fit of the model is reported by the coefficient of determination R^2^.

Associations between changes in gait characteristics (relative pre- to post changes) and changes in lower extremity strength (1-RM) respectively changes in functional performance (POMA) were quantified by Pearson’s correlation coefficients. Correlations were considered low (r < 0.2), moderate (r = 0.2-0.5), or good (r > 0.5) according to the recommendations of Cohen [38]. Data on intervention–related changes in lower extremity strength and functional performance (POMA) are not displayed in this paper since results have been published in a previous paper describing result of the parent study [20]. Statistical analysis was performed using SPSS statistics 17.0 (IBM, Armonk, NY, USA).

## Results

**Figure 1:**
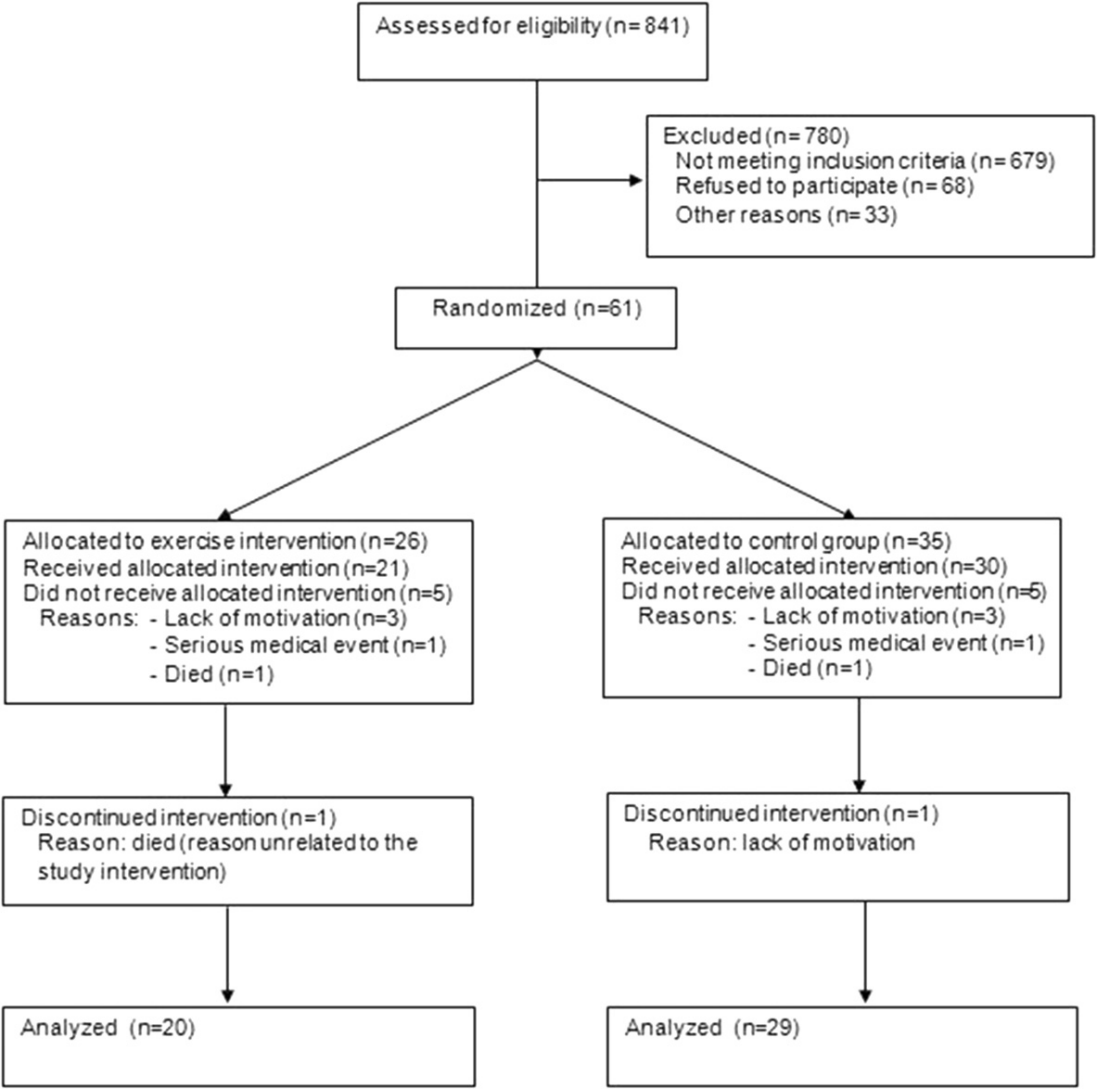
Flow diagram of progress through the phases of screening, enrolment, allocation, follow-up, and data analysis.

Eight hundred forty-one patients admitted to the hospital were screened for eligibility. The process of screening, enrolment, allocation, follow-up, and data analysis is shown in the Figure 1. Sixty-one subjects were recruited into the study. Ten individuals (16.4%) who had given consent and were randomized (5 IG, 5 CG) did not start the study after returning home from the hospital. Two participants (3.3%) dropped out during the intervention period (1 IG, 1 CG). Adherence to the intervention was excellent, averaging 91.9% in the IG and 94.4% in the CG. Training was safe despite participants’ advanced frailty, multimorbidity, and impairment, and no severe training-related adverse events occurred. No participant rejected the study challenges during training or testing.

The participants’ mean age was 81.9 ± 7.5 years and MMSE averaged 21.4 ± 2.9 points. Twenty-four (39.3%) had a possible depressive disorder based on the results of the screening tool (GDS ≥ 10 points). Baseline physical performance was low: Tinetti’s POMA averaged 20.7 ± 5.3 points and maximum gait speed averaged 1.3 ± 0.4 meters per second. Thirty-four (55.7%) subjects reported 1 or more falls in the last year. Fifty-four (88.5%) were discharged home after recruitment and 7 (11.5%) were institutionalized. No differences between IG and CG were found for any variable at baseline (Table 1). To control for correct randomization, baseline characteristics were compared between the IG and CG, considering only those participants who stayed in the trial until T2. No differences were found between dropout-adjusted groups (P = 0.101-0.934). Results are consistent with the statistical analysis for the whole group initially recruited (Table 1), suggesting no systematic bias by dropouts.

**Table 1:** Demographic and clinical characteristics of patients at baseline

**Characteristic**	**Intervention (n = 26)**	**Control (n = 35)**	**P-value**
Age, years	80.4 ± 7.1	82.3 ± 7.9	0.309
Women, number	17 (65.0)	22 (62.9)	0.839
Mini mental state examination, score	21.0 ± 2.9	21.7 ± 2.9	0.307
Education, years	11.3 ± 3.2	10.8 ± 2.3	0.462
Barthel activities of daily living, score	82.7 ± 17.5	83.2 ± 14.0	0.883
Geriatric depression scale 30 point, score	9.0 ± 5.9	8.6 ± 5.2	0.812
Cumulative illness rating scale, score	22.9 ± 3.1	22.8 ± 3.7	0.984
Number of medication	6.8 ± 3.4	6.4 ± 2.6	0.680
History of falls in the last year, number of patients	13 (50.0)	21 (60.0)	0.293
Performance oriented mobility assessment, score	21.1 ± 5.3	20.4 ± 5.4	0.630
Maximum lower extremity strength (1-RM leg press), KG	150.9 ± 57.5	143.0 ± 45.3	0.551
Maximum gait speed, m/sec	1.33 ± 0.54	1.28 ± 0.37	0.711

Data are mean ± standard deviation or number (%). 1-RM = one repetition maximum. P-values are given for differences between the intervention and control group.

### Effect of the intervention on spatio-temporal gait parameters

Values of baseline and follow-up assessment, adjusted mean treatment difference, effect sizes, and percentage change from baseline are reported in Table 2. Significant training-related improvements were obtained for gait speed, cadence, stride length, stride time and double support in the IG compared to the CG (P ≤ 0.001-0.008). Effect sizes were large for all gait parameters that changed significantly (range Cohen’s d: 0.80 – 1.27) with highest effects for gait speed and lowest effects for stride length. No significant effects were found for step width, step time variability, and Walk-Ratio (P = 0.554 - 0.999).

**Table 2:** Effect of the intervention on spatio-temporal gait variables

**Gait variables**	**Baseline IG: n = 20 CG: n = 29**	**Follow-up IG: n = 20 CG: n = 29**	**Mean treatment difference (95% CI)**^**a**^	**P-value**^**b **^**Effect size**^**c**^	**% change from baseline**^**d**^
Speed, cm/sec					
IG	132.67 ± 55.67	149.32 ± 48.21	18.32	<0.001	IG: 12.5
CG	128.66 ± 38.20	127.64 ± 35.65	(9.85 to 26.80)	1.27	CG: -0.8
Cadence, steps/min					
IG	137.14 ± 21.13	145.39 ± 20.82	11.16	0.002	IG: 6.0
CG	134.47 ± 17.85	131.98 ± 19.17	(4.37 to 17.94)	0.96	CG: -1.9
Stride length, cm					
IG	116.58 ± 42.55	124.82 ± 37.44	7.88	0.008	IG: 7.1
CG	115.31 ± 29.47	115.89 ± 25.66	(2.14 to 13.61)	0.80	CG: 0.5
Stride time, sec					
IG	0.90 ± 0.15	0.84 ± 0.13	-0.08	0.001	IG: 6.7
CG	0.91 ± 0.11	0.92 ± 0.12	(-0.12 to -0.03)	0.99	CG: -1.1
Double support,% of stride time					
IG	26.92 ± 8.93	23.04 ± 7.78	-2.89	0.001	IG: 14.4
CG	25.85 ± 6.06	25.39 ± 5.99	(-4.53 to -1.25)	1.03	CG: 1.8
Step width, cm					
IG	11.34 ± 4.18	11.08 ± 4.95	0.001	0.999	IG: 2.3
CG	10.18 ± 4.23	9.94 ± 4.42	(-1.24 to 1.24)	0.00	CG: 2.4
Step time variability, CV					
IG	5.24 ± 3.41	5.08 ± 2.05	-0.47	0.425	IG: 3.1
CG	5.03 ± 2.48	5.40 ± 2.56	(-1.65 to 0.71)	0.22	CG: -7.4
Walk-Ratio^e^					
IG	0.43 ± 0.16	0.44 ± 0.15	-0.01	0.554	IG: 2.3
CG	0.43 ± 0.12	0.44 ± 0.11	(-.04 to 0.02)	0.18	CG: 2.3

Data presented as mean ± standard deviation. IG, intervention group; CG, control group; CI, confidence interval; CV, coefficient of variation; ^a^adjusted for baseline value using ANCOVA (Baseline values total sample, n = 49: speed 130.30 ± 45.63; cadence 135.56 ± 19.09; stride length 115.82 ± 34.98; stride time 0.90 ± 0.13; double support 26.29 ± 7.30; step time variability 5.12 ± 2.88; step width 10.66 ± 4.21; walk-ratio 0.43 ± 0.14); ^b^P-values from ANCOVA comparing the effect of the intervention on gait parameters at follow-up adjusting for baseline values; ^c^adjusted mean treatment difference/pooled standard deviation; ^d^positive scores indicate improvement; ^e^step length/cadence ratio.

### Variables associated with improvement in gait characteristics

Predictors of improvement in gait speed were higher multimorbidity (CIRS: β = 0.554, R^2^ = 0.307, P = 0.011), higher depression (GDS: β = 0.460, R^2^ = 0.212, P = 0.041), and lower functional performance (POMA: β = -0.519, R^2^ = 0.269, P = .019) at baseline. In the multivariate regression model, higher multimorbidity (CIRS: β = 0.480, P = 0.011) and lower functional performance (POMA: β = -0.437, P = 0.023) at baseline were independently associated with improvements in gait speed (R^2^ = 0.493). Cognitive parameters (P = 0.191-0.725), age (P = 0.553), gender (P = 0.773), baseline strength (1-RM leg press, P = 0.190), and adherence (P = 0.624) did not predict training response.

Improvement in functional performance (POMA) was significantly associated with improvement in stride-length (r = 0.537, P = 0.015), but not with improvement in other gait variables (speed r = 0.400, P = 0.080; cadence r = 0.110, P = 0.646; stride time r = -0.152, P = 0.523; double support r = -0.080, P = 0.737). Improvement in lower extremity strength (1-RM leg press) was significantly associated with improvement in cadence (r = 0.454, P = 0.045), but not with improvement in other gait variables (speed r = 0.410, P = 0.073; stride length r = 0.257, P = 0.273; stride time r = -0.413, P = 0.071; double support r = -0.046, P = 0.847).

## Discussion

This study shows that a 12-week exercise program, based on progressive resistance and functional training, improved relevant spatio-temporal gait parameters in people with confirmed mild to moderate dementia. Present findings suggest that intensive exercise training is feasible in the target group and substantially improves functional performance in terms of walking ability, which is a hallmark of mobility-related quality of life and independence.

To prevent methodological shortcomings as reported in systematic reviews [14-17], a special focus was put on the design and intervention of the present study. Established, standardized training methods including high-intensity, progressive resistance and functional training were used that had been applied in successful interventions in multimorbid, frail older adults before [27,28], a dementia-specific, patient-centered approach supervised by trained instructors was developed, a clear case definition including confirmed diagnosis of dementia was used, a homogeneous sample with respect to cognitive status was selected and objective computerized spatio-temporal gait analysis was used to document training effects on gait characteristics. Predictors of training response that had not been addressed in previous studies were additionally reported on.

The adjusted mean treatment difference found for gait speed (18.3 cm/sec) represents a substantial clinically meaningful change [39,40]. Training effect on gait speed obtained in the present study considerably exceeded results of a recent meta-analysis of exercise effects on gait speed in people with dementia (mean difference: 6 cm/sec) [14].

Importantly, the exercise training improved both spatial (stride length) and temporal (double support) gait parameters which represent predictive indicators of fall-risk in people with dementia [41-43]. Reducing stride length as well as increasing the percentage of double support during the gait cycle is an attempt to minimize postural instability and indicates deficits in balance control [44]. Our results demonstrate that the stride length was significantly increased and the double support significantly reduced in the IG compared to the CG. These findings indicate improved dynamic balance control and reduced risk of falling during walking in people with dementia as a result of participating in the presented exercise training program.

Before starting the intervention spatio-temporal gait parameters of the study participants were comparable with those found in prefrail populations as described in a recent systematic review [45]. After the intervention gait characteristics in the IG were comparable with those of nonfrail older adults whereas gait performance in the CG remained on a prefrailty level. These results may suggest that physical prefrailty as characterized by gait impairment can be reversed in people with dementia using intensive progressive strength and functional training.

The gait improvements found in the present study are most likely related to the combination of resistance and functional exercises. Previous studies which used functional training (walking exercise) only in the target group did not report improvements in gait speed [46], potentially due to the lack of resistance exercises for improving lower extremity strength. Likewise, studies using resistance training only but no functional training also had limited effect on gait performances as reported in a systematic review [15]. Our study results show that improvements in gait characteristics are related to both a training related-increase in lower extremity strength and an increase functional performance (POMA). Present findings suggest that both resistance and functional training are important for improving gait performance in the target group.

While we found effects for several gait parameters as described above, other gait variables including step width, step time variability, and Walk-Ratio did not change as a result of the exercise program. Previous studies have identified an increase in step width as a compensation mechanism for gait instability in older adults [47]. Reduced stride width (which is similar to step width used in this study) has been linked to increased fall risk in older adults [48]. Specific exercise programs such as tai chi have resulted in increased stride width [49], potentially related to the tai chi characteristics including wide stances with large base of support. In contrast, our exercise program incorporated specific walking tasks such as narrowing step width aiming to improve lateral stability. However, these walking tasks did not result in a change of step width after the intervention, which may either suggest that participants did not improve in lateral stability or did not reduce step width intentionally as this may increase fall risk. It should be also noted that reliability of measuring step width is lower compared to measuring other spatio-temporal gait parameters such as step length [50], which may have masked potential training related changes in this parameter in the present study.

Increased gait variability has been defined as dementia-specific gait disorder not only associated to motor disorders but also to problems with central processing of information [51]. In the present study limited effects on step time variability may suggest that our exercise program is not effective for improving specific cognition-related gait characteristics but is rather effective for improving gait characteristics associated with strength/functional performances (i.e. gait speed, step length, cadence, etc.). It remains a future task if specific motor learning walking exercise programs including both overground and treadmill walking for reinforcing rhythmic stepping [52] are effective for reducing gait variability in people with dementia. On the same note, the short GaitRite® system (4.9 meter) used in the present study may have lowered reliability of measuring gait variability. Reliability of measuring gait variability increases as a function of walking distance and 20 meter continuous walking has been suggested for accurately measuring this parameter [53].

The Walk-Ratio in our sample at baseline (total group 0.43; female 0.41; male 0.47) was lower compared to physically active 80–85 years old women walking under same conditions (fast walking, Walk-Ratio 0.53 [35]), suggesting a ‘cautious gait pattern’ in our participants, as previously identified in individuals with dementia [54]. The Walk-Ratio decreases with increasing gait speed [35]. The finding in our study that there was no change in the Walk-Ratio following the trial may indicate that walking speed was increased, whereas the same walking stability was maintained, as discussed in earlier studies on the effect of exercise training on gait characteristics in non-demented older adults [55].

Cognitive status had been identified as a negative predictor of training response in some, but not all, observational studies [17]. In RCTs using general cognitive status for prediction of training response, effects of cognitive subdomains on trainability have rarely been studied. In the present study the general level of cognitive impairment and specific performance in cognitive subdomains did not influence training response. Our study results confirm that a response to exercise training can be achieved despite cognitive impairment.

Participants with higher multimorbidity and lower functional status at baseline had a better training response. These findings may suggest that the most functionally impaired participants reaped the most benefit. Results are in accordance with earlier studies indicating that participants with the lowest performance benefit most from physical activity interventions [56].

Based on the patient-centered approach, training adherence was excellent suggesting high feasibility of intensive exercise training specifically designed for individuals with dementia. Adherence exceeded the results of most other RCTs using exercise training in individuals with cognitive impairment [16,17]. Training was safe despite participants’ advanced frailty, multimorbidity, and impairment, and no severe training-related adverse events occurred.

A limitation of this study is that results achieved in participants with mild to moderate dementia may not generalize to those with more severe dementia. Although we developed a specific training program with high effectiveness for improvement on functional performances such as walking, we cannot exclude a time-related effect because time spent in group sessions differed between study groups. Another limitation is the lack of follow up data for quantifying the sustainability of intervention-related changes in gait characteristics.

## Conclusions

In summary, this RCT provides Class Ib evidence [57] that the presented, dementia-adjusted progressive strength and functional training regimen is beneficial for improving clinically relevant gait characteristics in people with mild to moderate dementia. The training program may represent a model for preventing and rehabilitating gait deficits in persons with dementia during early disease stage. Further research is required to identify exercise training programs effective for improving specific gait characteristics such as gait variability in people with dementia.

## Competing interests

The authors declare that they have no competing interests.

## Authors’ contributions

MS: Preparation of manuscript, statistical analysis, study management and interpretation of data. TZ: Acquisition of participants. SE: Statistical analysis. KH: Development of concept and design, study management, acquisition of funding. All authors including GG and BN contributed to interpretation of data, drafting the article and final approval of the version to be published.

## Pre-publication history

The pre-publication history for this paper can be accessed here:

http://www.biomedcentral.com/1471-2318/14/73/prepub
